# Changes in the medical-seeking pattern and daily behavior of hematopoietic stem-cell transplant recipients during the COVID-19 epidemic: An online survey in Hubei Province, China

**DOI:** 10.3389/fpubh.2022.918081

**Published:** 2022-10-04

**Authors:** Rong Xie, Yicheng Zhang, Zhiping Huang, Si Cheng, Jingming Guo, Youshan Zhang, Min Liu, Xiaojian Zhu, Yong You, Ping Zou, Wenlan Chen, Han Yan, Fanjun Cheng, Zhaodong Zhong

**Affiliations:** ^1^Institute of Hematology, Union Hospital, Tongji Medical College, Huazhong University of Science and Technology, Wuhan, China; ^2^Department of Hematology, Tongji Hospital, Tongji Medical College, Huazhong University of Science and Technology, Wuhan, China; ^3^Department of Hematology, Jingzhou Central Hospital, The Second Clinical Medical College, Yangtze University, Jingzhou, China; ^4^Department of Hematology, Yichang Central People's Hospital, The First College of Clinical Medical Science, China Three Gorges University, Yichang, China; ^5^Department of Hematology, Jingzhou First People's Hospital and First Affiliated Hospital of Yangtze University, Jingzhou, China

**Keywords:** hematopoietic stem-cell transplantation, medical-seeking pattern, daily behavior, COVID-19, lockdown

## Abstract

**Background:**

To curb the spread of the coronavirus disease 2019 (COVID-19) epidemic, the Chinese government shut down Wuhan city from January 23rd to April 8th, 2020. The COVID-19 epidemic not only leads to widespread illness but also affects the diagnosis and treatment of hematopoietic stem-cell transplant (HSCT) recipients.

**Objective:**

To investigate the medical-seeking pattern and daily behavior changes in Hubei Province during the COVID-19 epidemic in Hubei Province during the lockdown.

**Methods:**

We conducted a multicenter, cross-sectional, web-based investigation among 325 HSCT recipients by online questionnaires in Hubei Province during the COVID-19 epidemic.

**Results:**

A total of 145 complete responses were collected both before and during the epidemic questionnaires. The participants from pre-epidemic group preferred to go to hospital (68.29%) when they experienced influenza-like symptoms. The majority of the patients elected to take oral drugs by themselves (40%) or consulted their attending physicians online or by telephone during the lockdown (23.33%). 64.83% had difficulties in purchasing drugs during the lockdown, which was significantly higher than the proportion of the pre-epidemic group (24.83%) (*P* < 0.05). The participants preferred to purchase drugs online (23.40%) and decrease or withdraw drugs (18.09%) during the epidemic. The number of participants received regular re-examinations during the epidemic decreased sharply. The proportion of wearing masks and isolating themselves at home increased significantly during the epidemic. No statistic difference was observed in the incidence of graft-versus-host disease (GVHD)complications in participants between the during the epidemic group and the pre-epidemic group. In our study, six patients were confirmed to have COVID-19, and half of them died due to COVID-19-related complications.

**Conclusion:**

The medical-seeking pattern and daily behavior of HSCT recipients changed during the lockdown; the methods of self-protection, online consultation and drug delivery can help patients receive necessary follow-up and reduce the occurrence of COVID-19.

## Introduction

To curb the spread of the coronavirus disease 2019 (COVID-19) epidemic, the Chinese government shut down Wuhan city from January 23rd to April 8th, 2020 (1, 2). Transportation within Wuhan city and transportation between Wuhan city and other cities in Hubei Province was highly restricted. To date, the COVID-19 vaccine has not been administered to the entire population, and thus, severe acute respiratory syndrome coronavirus 2 (SARS-CoV-2) may be lead to another pandemic (3, 4). Areas of unmet need and future research questions are also highlighted. The waves of the COVID-19 pandemic are predicted to be followed by an “endemic” phase and therefore an ongoing risk within a “new normality” (5).

COVID-19 not only leads to widespread illness but also affects the diagnosis and treatment of patients with other diseases due to strict prevention and control strategies. Among these patients, hematopoietic stem-cell transplant (HSCT) recipients are likely to be seriously affected (6–8). Due to the severity of the primary disease and the development of immunosuppression after transplantation, recipients of HSCT are constantly at risk for serious complications such as graft-versus-host disease (GVHD), pulmonary infection, and relapse of primary disease (8, 9). Regular follow-up and re-examination can directly determine the prognosis of HSCT recipients. Thus, how doctors treat HSCT recipients and subsequent complications during the lockdown have become two crucial problems.

Leading global institutions, such as the European Society for Blood and Marrow Transplantation (EBMT) and American Society for Transplantation and Cellular Therapy (ASTCT), have published guidelines for COVID-19 management among HSCT recipients (9, 10). The EBMT recommends that potential transplant recipients should home isolate for 14 days pretransplant (9). The EBMT and the ASTCT recommend that transplant-related procedures be delayed 14–21 days from the last contact and potential recipients be monitored for SARS-CoV-2 infection in patients if they had come in close contact to persons infected with SARS-CoV-2 (9, 10). A major transplant center in China suggested that social distancing, mask wearing, and education for patients and donors are essential preventative measures in these patients (11). They also recommend potential transplant recipients and donors to finish SARS-CoV-2 with qRT-PCR, serum antibody detection, and a lung CT scan pretransplant (11).

Data about medical-seeking pattern and daily behavior changes during lockdown are still rare. Therefore, we performed a retrospective multicenter investigation to explore the changes in the medical-seeking pattern and daily behavior of HSCT recipients, and the incidence and mortality of HSCT recipients with COVID-19 in Hubei Province during lockdown.

## Methods

### Study participants and survey design

We conducted a multicenter, cross-sectional, web-based investigation among HSCT recipients using online questionnaires. This survey was conducted from 25 April to 30 May 2020. Patients were recruited from Hubei Province. The physicians used WeChat (the most popular social app in China) and DoctorWith (an online medical app used by HSCT recipients) to publicize and distribute the survey link to the patients or their direct relatives.

Respondents were informed that their participation was voluntary, and consent was implied on the completion of the questionnaire. The inclusion criteria were that the respondents were at least 18 years old, could read and comprehend Chinese, and were under travel restrictions. This study was approved by the Ethics Committee of Tongji Medical College, Huazhong University of Science and Technology, Wuhan, China (Approval: 2022-S078).

### Data collection

The research team develop two questionnaires to investigate HSCT recipients' medical-seeking pattern and daily behavior changes during the COVID-19 epidemic. Participants were recruited through multiple cities online panel providers for each city to avoid coverage bias: ZDZ and YCZ provided 157 responsers across Wuhan city; YSZ, JMG, and ZPH provided 109 responders from 3 cities in Hubei province except Wuhan. Respondents' identities were verified using IP addresses and mobile phone numbers to ensure that each participant was real and unique upon initial registration.

This survey included questions that assessed: (1) demographic background, the diagnosis of primary disease, HSCT complications; (2) self-perceived health status, daily behavior, medical-seeking pattern; (3) COVID-19 experience, the difficulties in suffering uncomfortable symptoms, the most fear during the COVID-19 epidemic.

Two online questionnaires (Supplement 1) were distributed and collected by physicians from each center. The first questionnaire investigated the medical-seeking pattern and daily behavior during COVID-19 epidemic, and the second questionnaire assessed the patient's routine before the COVID-19 epidemic. The questionnaire included 28 questions that assessed: (1) demographic characteristics, primary disease information, HSCT complications; (2) self-perceived health status, diagnosis and treatment modes, daily behavior, (3) COVID-19 experience, the difficulties in suffering uncomfortable symptoms, the most fear during the COVID-19 epidemic diagnosis, treatment regimen and mental stress during and before the COVID-19 epidemic.

The diagnosis of COVID-19 was made according to the diagnostic and treatment guidelines for COVID-19 issued by the Chinese National Health Committee (version 3–5). Two physicians independently reviewed the forms to verify the data. If data were not available from the questionnaire, the researchers directly communicated with the patients, health-care providers and/or patients' families to ascertain epidemiology and symptoms.

### Statistical analysis

For the HSCT recipients, data on HSCT related complications, and behavior patterns were expressed as descriptive statistics; continuous variables were expressed as medians and interquartile ranges (IQRs); and categorical variables were expressed as proportions and frequencies. Medians were compared using independent samples *t*-tests when the data were normally distributed; otherwise, the Mann-Whitney test or Kruskal-Wallis test was used. Proportions for categorical variables were compared using the χ2 test, although the Fisher's exact test was used when the data were limited. A *P* value of <0.05 was considered statistically significant. The analysis was completed in GraphPad Prism software, version 8.0.

## Results

### Characteristics of HSCT recipients during the COVID-19 epidemic

**Figure 1:**
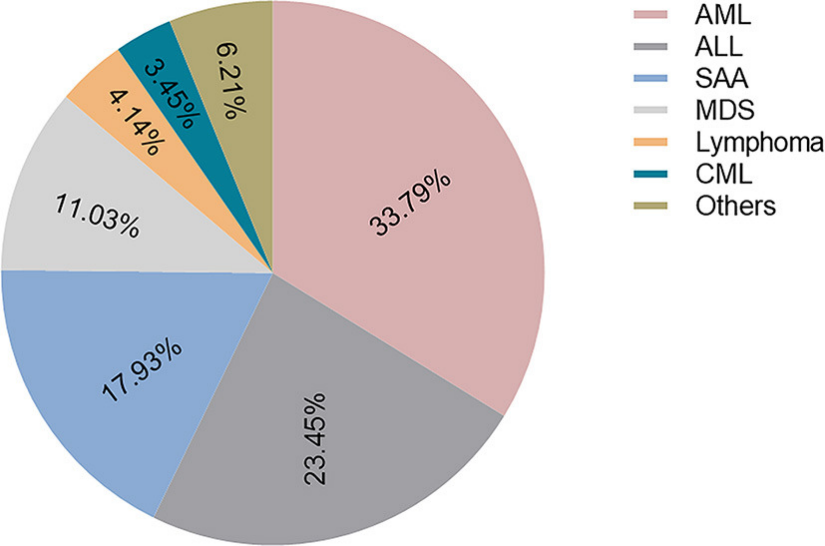
The diagnosis of primary disease. AML, Acute myelocytic leukemia; ALL, Acute lymphoblastic leukemia; SAA, Severe aplastic anemia; MDS, Myelodysplastic syndrome; CML, Chronic myeloid leukemia.

A total of 501 complete responses from 325 HSCT recipients were received, including 228 responses before the COVID-19 outbreak (from October 2019 to December 2019) and 273 during the epidemic (from February 2020 to April 2020). The overall response to the survey was poor. Only 145 participants returned both questionnaires at the same time. The demographics and baseline characteristics of the HSCT recipients are shown in Table 1 and Figure 1. Of note, all of the cohorts comprised HSCT recipients from Hubei Province, because of the largest number of confirmed cases in Hubei Province. The median age of the participants was 33 (23–41, IQR) years. A higher proportion of participants from other cities in in Hubei Province except Wuhan (66.21%) than Wuhan city (22.07%) and other provinces (11.72%) responded to our questionnaires. Figure 1 shows the diagnosis of primary diseases of responses. The great majority had matched sibling donors (97.93%) compared to matched unrelated donors (2.07%).

**Table 1:** Characteristics of hematopoietic stem cell transplantation recipients during the COVID-19 epidemic.

**Characteristics (*N* = 145)**	
**Age, median (IQR), y**	33 (23–41)
**Male/Female**	82/63
**Residence**	
Wuhan city	32 (22.07)
Other cities in Hubei province except Wuhan	96 (66.21)
Outside the Hubei province	17 (11.72)
**Primary disease**	
AML	49 (33.79)
ALL	34 (23.45)
SAA	26 (17.93)
MDS	16 (11.03)
Lymphoma	6 (4.14)
CML	5 (3.45)
Others	9 (6.21)
**Median time of transplantation, (IQR), M**	22.20 (11.35–37.17)
**Type of donor**	
Allo-HSCT	145 (100.00)
Sibling donors	142 (97.93)
Unrelated donor	3 (2.07)
**HLA matching of cell graft between donor and recipient**
Fully matched	48 (33.10)
Partially matched	59 (40.69)
Haploidentical	38 (26.21)

Data are presented as n (%) unless indicated otherwise. COVID-19, Coronavirus disease 2019; IQR, Interquartile range; AML, Acute myelocytic leukemia; ALL, Acute lymphoblastic leukemia; SAA, Severe aplastic anemia; MDS, Myelodysplastic syndrome; CML, Chronic myeloid leukemia; HSCT, Hematopoietic stem cell transplantation; HLA, Human leukocyte antigen.

### HSCT complications during the COVID-19 epidemic

Interestingly, no statistic difference was observed in the incidence of GVHD in HSCT recipients between the during the epidemic group (35.17%) and the before group (37.24%) (*P* = 0.81). The involved organs include skin, mouth, liver, joints and fascia, gastrointestinal, lungs, muscle, and eyes. Each of involved organ had similar incidence of GVHD between these two groups. In addition, only one patient experienced relapse of the primary disease during epidemic. The number of patients developed a pulmonary infection during the epidemic (5.52%) had no statistic difference in the pre-epidemic group (10.34%) (*P* = 0.19). Data on complications among HSCT recipients are shown in Table 2.

**Table 2:** HSCT complications of HSCT recipients during the COVID-19 epidemic.

**Complications** **(*N* = 145)**	**Before the epidemic**	**During the epidemic**	***P* Value**
**GVHD**	54 (37.24)	51 (35.17)	0.81
Skin	35 (24.14)	27 (18.62)	0.32
Mouth	14 (9.66)	20 (13.79)	0.36
Liver	13 (8.97)	14 (9.66)	>0.99
Joints and fascia	10 (6.90)	11 (7.59)	>0.99
Gastrointestinal	7 (4.83)	4 (2.76)	0.54
Lungs	9 (6.21)	8 (5.52)	>0.99
Muscle	8 (5.52)	4 (2.76)	0.38
Eyes	0	2 (1.38)	0.50
**Relapse**	0	1 (0.69)	>0.99
**Pulmonary infection**	15 (10.34)	8 (5.52)	0.19

Data are presented as n/N (%) unless indicated otherwise. COVID-19, Coronavirus disease 2019; HSCT, Hematopoietic stem cell transplantation; GVHD, Graft-versus-host disease.

### Medical treatments and daily behavior patterns of HSCT recipients

**Figure 2:**
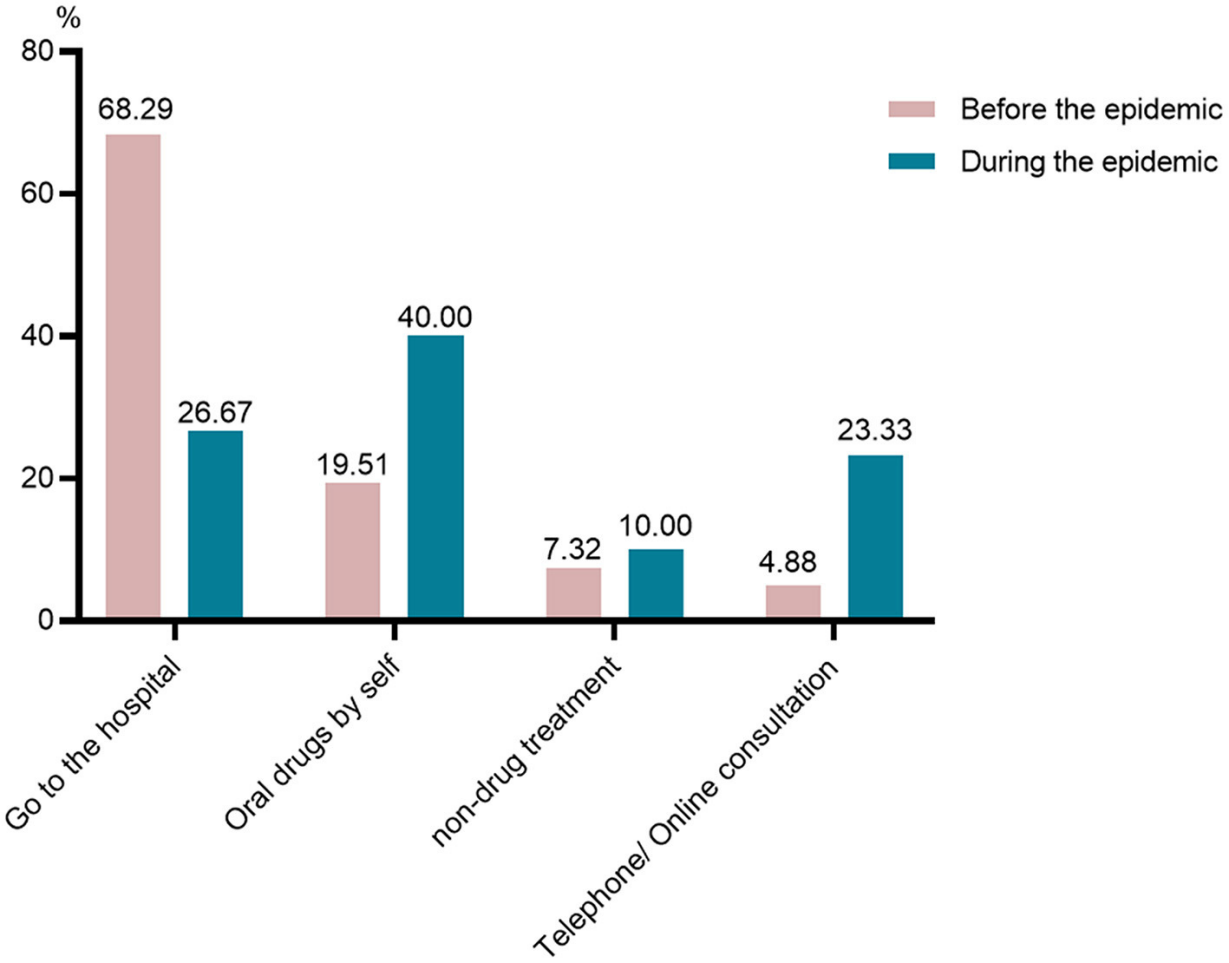
Different solutions for influenza-like symptoms.

Our study found that the number of participants with cough or fever did not increase during the epidemic. The proportion of participants with influenza-like symptoms was 20.69% during the epidemic and 28.28% before the epidemic (*P* = 0.17). Although the number of participants experienced influenza-like symptoms did not increase during the epidemic, they differ in solutions for these uncomfortable symptoms (Figure 2). The participants from pre-epidemic group preferred to go to hospital (68.29%) when they experienced cough or fever. However, 40% of the patients elected to take oral drugs by themselves during the lockdown. What can be clearly seen in Figure 2 is the growth of the number of patients consulted their attending physicians online or by telephone during the epidemic (23.33%).

**Figure 3:**
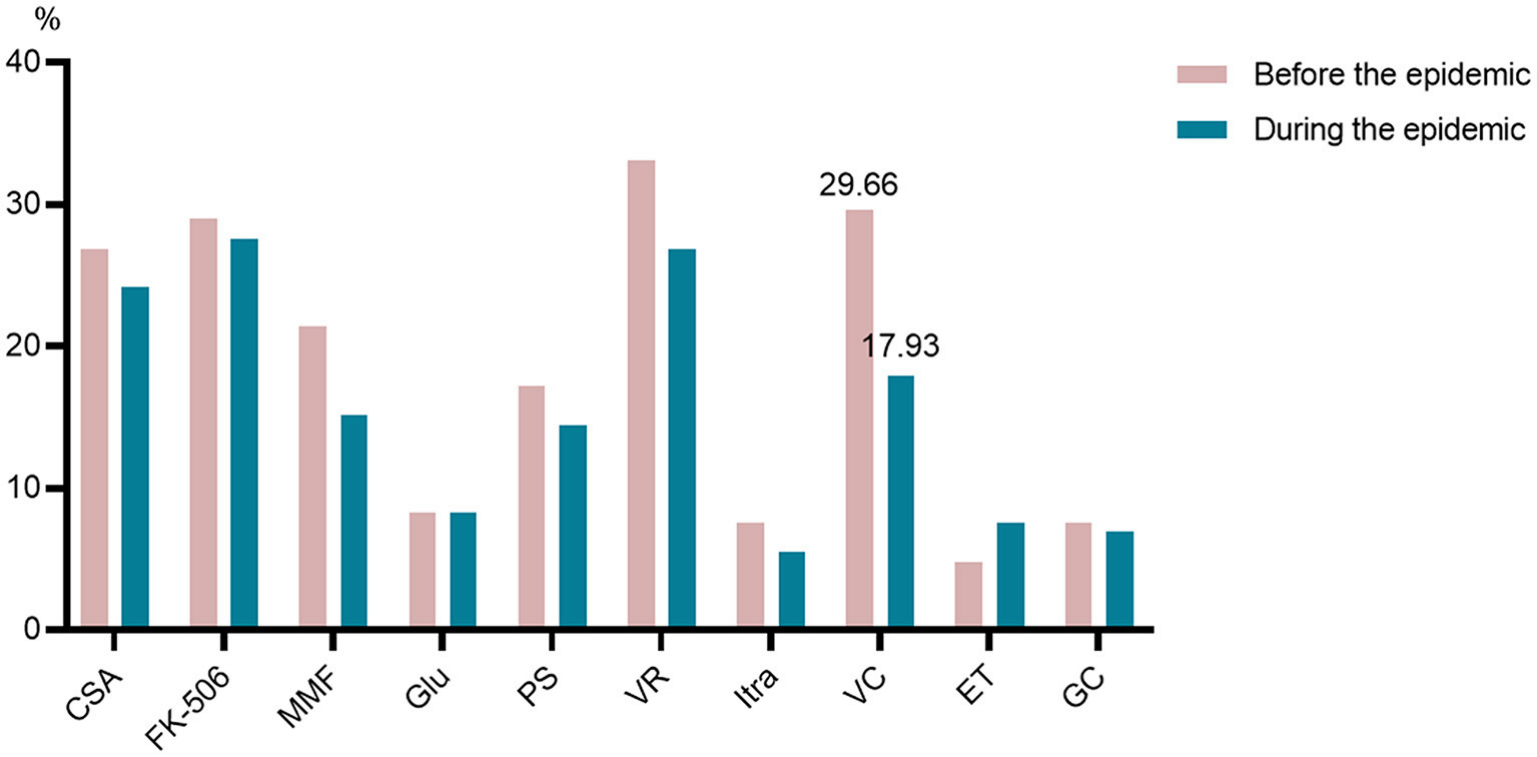
Routine medicines after hematopoietic stem-cell transplantation. CSA, cyclosporine a; FK-506, tacrolimus; MMF, mycophenolate mofetil; GLU, glucocorticoids; PS, posaconazole; VR, voriconazole; Itra, itraconazole; GC, garlicin capsules; VC, valaciclovir; ET, entecavir.

The participants received routine therapy after hematopoietic stem-cell transplantation, including immunosuppressive therapy (cyclosporine a, or tacrolimus, and/or mycophenolate mofetil, or glucocorticoids), antifungal therapy (posaconazole, or voriconazole, or itraconazole, and/or garlicin capsules), antiviral therapy (valaciclovir, and/or entecavir), micromolecule targeted medicines (imatinib, or dasatinib, or ruxolitinib, or ibrutinib). The number of patients on medicines were shown in the Table 3. We found that of 145 participants, 64.83% had difficulties in purchasing drugs during the lockdown, which was significantly higher than the proportion of the pre-epidemic group (24.83%) (*P* < 0.05). The evidence shows that the participants preferred to purchase drugs online (23.40%) and decrease or withdraw drugs (18.09%) during the epidemic. And patients in the pre-epidemic group preferred borrow drugs from wardmate group (25.00%) and decrease or withdraw drugs (16.67%) when they were hard to purchasing medicines. What stands out in Figure 3 is the dramatic decline in the number of patients on valaciclovir after the lockdown.

**Table 3:** The influences of the COVID-19 epidemic on medical treatments and daily behavior patterns.

**Characteristics** **(*N* = 145)**	**Before the epidemic**	**During the epidemic**	***P* Value**
**Symptoms of flu**	41 (28.28)	30 (20.69)	0.17
Cough	33 (22.76)	25 (17.24)	0.30
Fever	19 (13.10)	10 (6.90)	0.12
**Solutions for the uncomfortable symptoms**			
Go to the hospital	28/41 (68.29)	8/30 (26.67)	<0.05
Oral drugs by self	8/41 (19.51)	12/30 (40.00)	0.07
non-drug treatment	3/41 (7.32)	3/30 (10.00)	0.69
Telephone/ online consultation	2/41 (4.88)	7/30 (23.33)	<0.05
**Routine therapy after HSCT**			
Cyclosporine a	39 (26.90)	35 (24.14)	0.69
Tacrolimus	42 (28.97)	40 (27.59)	0.90
Mycophenolate mofetil	31 (21.38)	22 (15.17)	0.22
Glucocorticoids	12 (8.28)	12 (8.28)	>0.99
Posaconazole	25 (17.24)	21(14.48)	0.63
Voriconazole	48 (33.10)	39 (26.90)	0.31
Itraconazole	11 (7.59)	8 (5.52)	0.64
Garlicin capsule	11 (7.59)	10 (6.90)	>0.99
Valaciclovir	43 (29.66)	26 (17.93)	<0.05
Entecavir	7 (4.83)	11(7.59)	0.47
Imatinib	6 (4.14)	6 (4.14)	>0.99
Dasatinib	3 (2.07)	3 (2.07)	>0.99
Ruxolitinib	16(11.03)	13(8.97)	0.70
Ibrutinib	4 (2.76)	3 (2.07)	>0.99
**Difficulties in purchasing drugs**	36 (24.83)	94 (64.83)	<0.05
**Solutions for the difficulties**			
Purchasing drugs by volunteers	5/36 (13.89)	10/94 (10.64)	0.56
Purchasing drugs online	4/36 (11.11)	22/94 (23.40)	0.15
Borrow drugs from wardmate group	9/36 (25.00)	14/94 (14.89)	0.20
Purchasing drugs in pharmacy	4/36 (11.11)	8/94 (8.51)	0.74
Seek help from local government departments	2/36 (5.56)	11/94 (11.70)	0.51
Purchasing drugs in hospital	5/36 (13.89)	7/94 (7.45)	0.31
Use similar drugs	1/36 (2.78)	2/94 (2.13)	>0.99
Drugs decrement or withdrawal	6/36 (16.67)	17/94 (18.09)	>0.99
Others	1/36 (2.70)	6/94 (6.38)	0.67
**Regular re-examinations**			
Blood routine	127 (87.59)	94 (64.83)	<0.05
Biochemical function	114 (78.62)	74 (51.03)	<0.05
Bone marrow aspiration	63 (43.45)	12 (8.28)	<0.05
Serum drug concentration	52 (35.86)	20 (13.79)	<0.05
**Wearing masks**	116 (80.00)	141 (97.24)	<0.05
**Going outdoors**	72 (49.66)	24 (16.55)	<0.05
**Living in a separate room**	101 (69.66)	122 (84.14)	<0.05

Data are presented as n/N (%) unless indicated otherwise. COVID-19, Coronavirus disease 2019.

**Figure 4:**
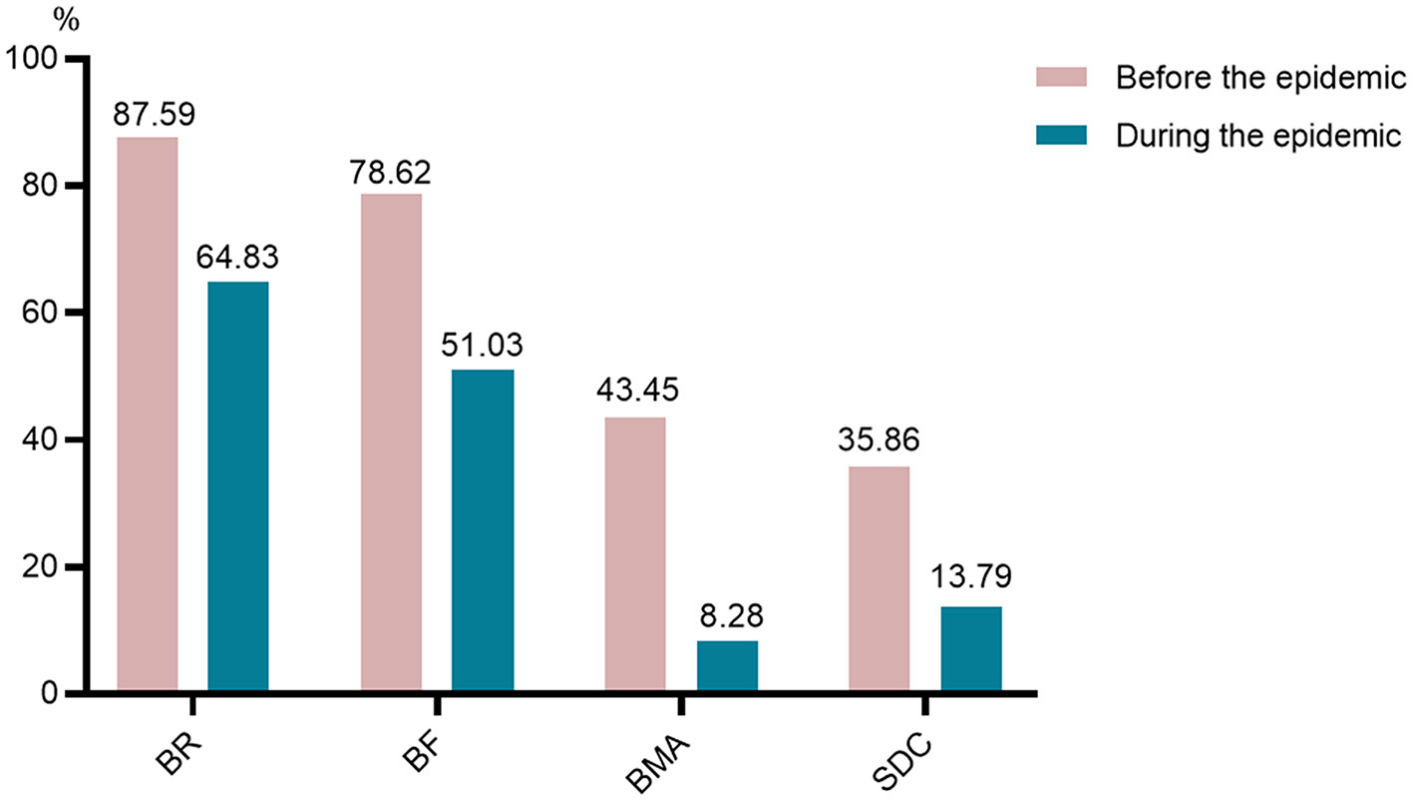
Changes of regular re-examinations. BR, blood routine; BF, biochemical function; BMA, bone marrow aspiration; SDC, serum drug concentration of cyclosporine or tacrolimus.

**Figure 5:**
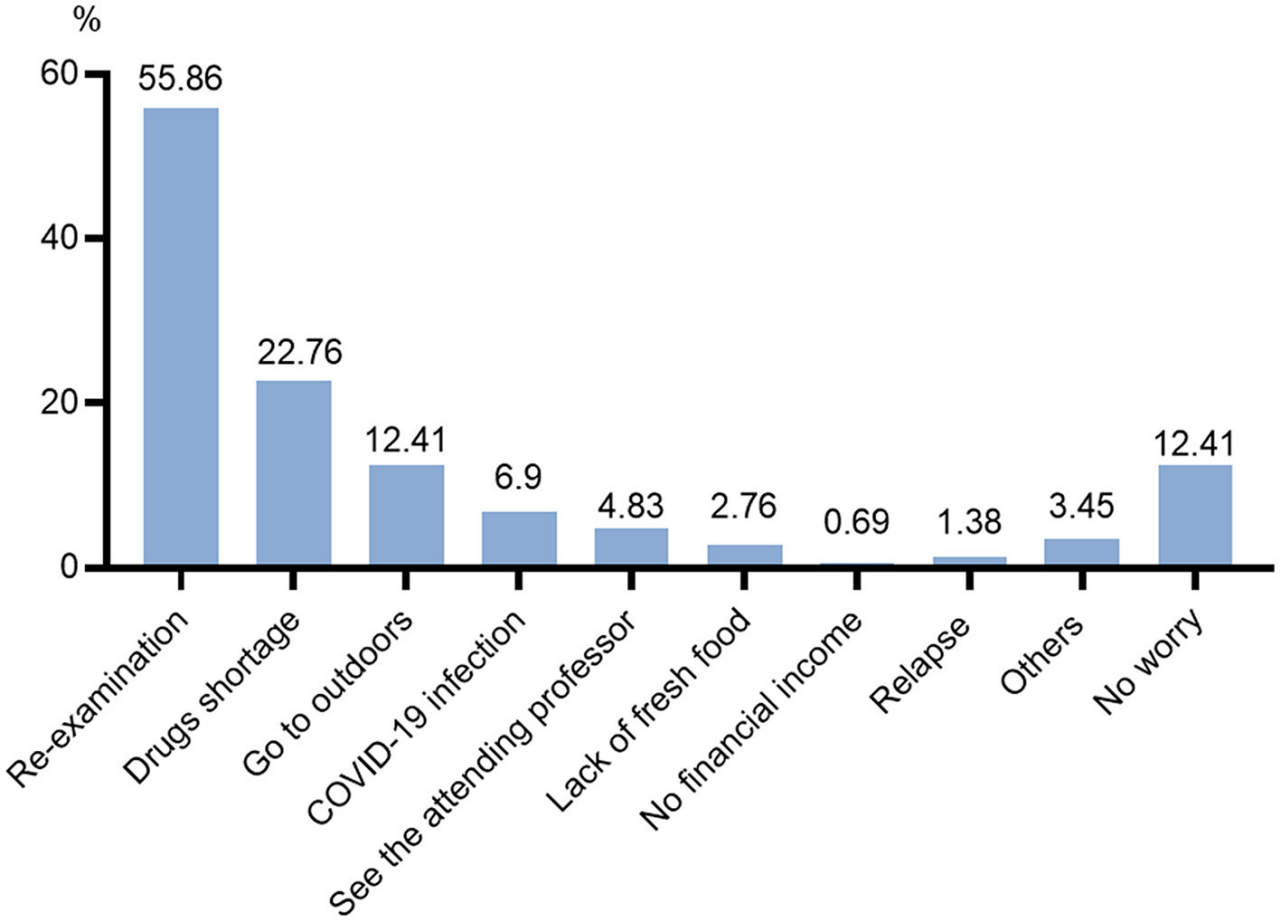
The biggest fear of the participants during the COVID-19 epidemic.

Figure 4 and Table 3 reveals that there has been a sharp drop in the number of participants received regular re-examinations during the epidemic, whether blood routine, biochemical function, bone marrow aspiration, or serum drug concentration of cyclosporine or tacrolimus. Furthermore, this is the biggest fear of the participants during the epidemic (Figure 5). With the lockdown, the probability of wearing masks increased by 17.24% (*P* < 0.05). Meanwhile, the proportion of going outdoors reached 16.55% during the epidemic far fewer the pre-epidemic group (49.66%) (*P* < 0.05). Also, more participants lived in a separate room (84.14%) during the lockdown than before (69.66%) (*P* < 0.05).

### Characteristics of patients with COVID-19 after HSCT

In our study, six patients were confirmed to have COVID-19, which was not higher than that in the general population (12, 13). Their clinical features and auxiliary examinations are shown in Table 4. Four patients lived in Wuhan, and two had sojourn in Wuhan city. All confirmed patients had a fever. Their chest CT scan showed that almost all of them had bilateral lung infections (5/6). SARS-CoV-2 nucleic acid was detected in 3 patients. The antibody test was performed in 4 patients: three of them were IgM positive, and all were IgG negative. In total, three patients died during the pandemic, the leading cause of death were multiple organ dysfunction syndrome (1/3), infectious shock (1/3), cerebral hemorrhage (1/3), and respiratory and circulatory failure (2/3).

**Table 4:** Characteristics of patients with COVID-19 after HSCT.

**Characteristics**	**Confirmed patients**					
	**01 (Tian)**	**02 (Xing)**	**03 (Bai)**	**04 (Ding)**	**05 (Luo)**	**06 (Hong)**
**Age, y**	20	17	15	24	24	42
**Sex**	Male	Female	Male	Female	Male	Female
**Primary disease**	ALL	AML	AML	PMBL	AML	AML
**Home**	Wuhan	Wuhan	Hubei	Wuhan	Wuhan	Hubei
**Time from allo-HSCT to COVID-19, M**	15	15	11	4	11	3
**Donor**	Mother	Father	Brother	Auto-HSCT	Brother	Brother
**Signs and symptoms**						
Fever	Yes	Yes	Yes	Yes	Yes	Yes
Cough	Yes	Yes	No	No	No	No
Expectoration	Yes	Yes	No	No	No	No
Dyspnea	No	Yes	No	No	No	No
Diarrhea	Yes	No	No	No	No	No
Sore throat	No	No	No	No	No	Yes
**Chest CT image**						
Single/ Bilateral lung	Bilateral	Bilateral	Single	Bilateral	Bilateral	Bilateral
Dim patch	Yes	Yes	No	Yes	No	No
Stripe shadow	No	No	No	No	No	Yes
Ground glass opacity	Yes	Yes	No	No	Yes	No
Nodule	No	No	Yes	No	No	No
Pleural effusion	No	No	No	Yes	Yes	No
**SARS-CoV-2 nucleic acid detection**	Positive	Weak-positive	Negative	Positive	Negative	Negative
**SARS-CoV-2 antibody test**
IgG	NA	Negative	Negative	NA	Negative	Negative
IgM	NA	Negative	Positive	NA	Positive	Positive
**Outcome of patients**	Deceased	Cured	Deceased	Deceased	Cured	Cured
**Complications**						
Acute respiratory distress syndrome	No	Yes	Yes	No	No	No
Shock	Yes	No	Yes	Yes	No	No
Kidney injury	Yes	No	Yes	No	No	No
Liver injury	Yes	Yes	Yes	Yes	Yes	No
CNS injury	No	No	No	Yes	No	No
**Cause of death**	Multiple organ dysfunction syndrome	NA	Infectious shock, respiratory failure	Cerebral hemorrhage respiratory and circulatory failure	NA	NA

COVID-19, Coronavirus disease 2019; HSCT, Hematopoietic stem cell transplantation; AML, Acute myelocytic leukemia; ALL, Acute lymphoblastic leukemia; PMBL, Primary mediastinal B-cell lymphoma; IgG, Immunoglobulin G; IgM, Immunoglobulin M; CNS, Central nervous system.

## Discussion

To our knowledge, this is the first published survey of HSCT recipients whose changes in medical-seeking pattern and daily behavior patterns before and during the COVID-19 epidemic. We observed the majority of participants had medical-seeking pattern and daily behavior patterns changes during the lockdown. The survey shows that there has been a marked increase in the number of patients wearing masks and living a separate room during epidemic. Meanwhile, participants have to travel less and better insulate their homes due to the lockdown. Compared with participants in pre-epidemic period, participants in epidemic changed their medical-seeking pattern and purchasing medicine mode. The participants stay alert to influenza-like symptoms all the time, but in contrast to go to hospital immediately when they had a fever or cough, seek medical advices from a distance was an alternative choice.

To enhance the awareness of COVID-19 prevention among HSCT recipients, we strengthened patient education and psychological counseling through online platforms during the lockdown. We also provided consultations and advices to patients online. Therefore, HSCT recipients paid more attention to personal protection, they preferred to wear masks; and they refrained from going outdoors. As a result, only 6 of 375 HSCT recipients experienced SARS-CoV-2 infections. In addition, our study showed that the recommendation to wear masks and limit going outdoors may reduce the risk of SARS-CoV-2 infection. This conclusion was consistent with Bundgaard.H's research (14).

Prior to this survey, there has only been a small amounts of published articles describing how to manage hematopoietic cell transplant during the SARS-CoV-2 pandemic (11, 15–19). They reported that patients received HSCT are at increased risk of infection with SARS-CoV-2 and COVID-19 (18). A major transplant center in China recommended that self-isolation and minimal contact with family members, and nonessential clinic visits should be deferred or substituted with telemedicine consultations if possible (11).

Based on a patient's specific adverse effects to HSCT and his/her uncomfortable symptoms, an individualized stratified diagnosis and treatment plan is programmed online through WeChat or DoctorWith software. The incidence of HSCT complications such as GVHD, infection, and relapse did not increase during the pandemic, which may suggest that seeking medical advice online is a useful strategy for the management of HSCT recipients and other chronic diseases. The difficulty of purchasing drugs during the lockdown maybe the important factor for drugs decrement or withdrawal. In this study, approximately a quarter of those who are short of medicines appealed to wardmate group or volunteers to borrow or purchase drugs and send them directly. We hypothesized that these wardmates and volunteers may have helped the patients with purchasing drugs before the epidemic.

This study has several limitations. First, the number of patients responded to the survey is small; however, it parallels the size of other studies that have had a significant influence in the field of HSCT recipients (18, 20). Despite the small size of this survey, it was representatively in medical-seeking pattern and daily behavior patterns changes of HSCT recipients during the lockdown. Therefore, a larger cohort is required to ascertain whether these initial findings are transferable to specialized at-risk cohorts. In contrast to other patients, HSCT recipients are more likely to develop opportunistic infections due to impaired immunity (12, 21). In addition, GVHD, infection, and relapse usually occur sequentially in patients after transplantation, and are the leading causes of premature death (21–24). There were insufficient severe HSCT complications to provide a conclusive evaluation of the changes of incidence and severity of complications during the epidemic.

The other limitation is that the participants who obtained and completed the link of questionnaires aged 18–60 years were overrepresented, which may lead to selection bias. All of participants are HSCT recipients who were younger than the general population (25–27), one of the main reasons is the age limitation of HSCT, also due to the primary user community of WeChat or DoctorWith are younger and middle-aged. In addition, the diagnosis of COVID-19 was made according to the diagnostic and treatment guidelines for COVID-19 issued by the Chinese National Health Committee (version 3–5) in the early epidemic. Therefore, many COVID-19 patients were empirically diagnosed and lacked a molecular diagnosis. Also, the RT-PCR kits in use (likely the first kits developed for SARS-CoV-2 detection), the lack of standard methods for COVID-19 testing may lead to false negative nucleic acid results (28–30). Finally, we relied on several patient families completed questionnaires in special patients, and thus, blinding to the intervention was not possible.

In conclusion, in light of their changes in medical-seeking pattern and daily behavior patterns during the lockdown, online consultation and drug delivery can help patients receive necessary follow-up and reduce the occurrence of adverse events. Further studies would need to evaluate the availability of telemedicine in the management of HSCT recipients under the normalization of the epidemic.

## Data availability statement

The original contributions presented in the study are included in the article/Supplementary material, further inquiries can be directed to the corresponding authors.

## Ethics statement

The studies involving human participants were reviewed and approved by Ethics Committee of Tongji Medical College, Huazhong University of Science and Technology. Respondents were informed that their participation was voluntary and written informed consent was obtained to participate in this study.

## Author contributions

ZZ, FC, and YiZ designed the research. ZH, SC, JG, YoZ, ML, WC, and HY collected the data. RX and XZ analyzed results and wrote the paper. ZZ, YY, PZ, and FC interpreted data and reviewed the paper. All authors reviewed the results and approved the final version of the manuscript.

## Conflict of interest

The authors declare that the research was conducted in the absence of any commercial or financial relationships that could be construed as a potential conflict of interest.

## Publisher's note

All claims expressed in this article are solely those of the authors and do not necessarily represent those of their affiliated organizations, or those of the publisher, the editors and the reviewers. Any product that may be evaluated in this article, or claim that may be made by its manufacturer, is not guaranteed or endorsed by the publisher.
