# Supplementary material for: Changes in the medical-seeking pattern and daily behavior of hematopoietic stem-cell transplant recipients during the COVID-19 epidemic: An online survey in Hubei Province, China
Source: Front Public Health. 2022 Oct 4;10:918081. doi: 10.3389/fpubh.2022.918081 (PMC9577240; doi:10.3389/fpubh.2022.918081)
Supplement: Supplementary file 1 [file Data_Sheet_1.pdf]

Questionnaire on hematopoietic stem cell transplantation recipients during COVID-19 epidemic in Hubei province

Q1. What is your name?

Q2. How old are you?

Q3. What is your gender?

A.Male

B.Female

Q4. Where did you live during the coronavirus disease 2019 (COVID-19) epidemic?

A.Wuhan city

B.Other cities in Hubei Province except Wuhan

C.Outside the Hubei province

Q5. What is your telephone number?

Q6. What is your primary disease?

A. Acute myeloid leukemia(AML)

B. Acute lymphoblastic leukemia(ALL)

C. Severe aplastic anemia(SAA)

D. Myelodysplastic syndrome(MDS)

E. Lymphoma

F. Chronic myeloid leukemia(CML)

G. Multiple myeloma(MM)

H. Others

Q7. Which blood group did you have before the hematopoietic stem cell transplantation (HSCT)?

Q8. When did you get the HSCT?

Q9. Which type of HSCT did you get?

A. Allogenic hematopoietic stem cell transplantation (Allo-HSCT)

B. Autologous hematopoietic stem cell transplantation (Auto-HSCT)

Q10. What is the relationship between the donor and you?

A. Parents

B. Brothers or sisters

C. Children

D. Unrelated donor (from Chinese Bone Marrow bank)

Q11. Which blood group did you have after the HSCT?

Q12. How many points in HLA- matching did you have?

Q13. Did you have graft-versus-host disease(GVHD) during the epidemic?

A. Yes
B. No

Q14. If you had GVHD, which organs were involved? (Multiple choices)

A. Skin
B. Mouth
C. Lungs
D. Liver
E. Gastrointestinal
F. Joints and fascia
G. Muscle
H. Eyes
I. Others

Q15. Did you have a relapse of the primary disease during the epidemic?

A. Yes
B. No

Q16. Did you go out during the epidemic?

A. Yes
B. No

Q17. Did you wear mask consciously during the epidemic?

A. Yes
B. No

Q18. Did you live in a separate room during the epidemic?

A. Yes
B. No

Q19. Did you have the following symptoms during the epidemic? (Multiple choices)

A. Fever
B. Cough
C. Pulmonary infection (chest image)

Q20. When you had above symptoms, what did you do during the epidemic?

A. Go to the hospital
B. Take oral drugs by yourself
C. Consult doctor online/ telephone
D. None

Q21. Did you have difficulty in buying oral drugs during the epidemic?

A. Yes
B. No

Q22. How did you solve the difficulty in buying drugs during the epidemic?

A. Look for volunteers to buy drugs
B. Reduce the dose of oral drugs or stopping taking medicine
C. Buy drugs online
D. Borrow drugs from other patients
E. Buy drugs in pharmacies
F. Seek help from local government departments
G. Buy drugs in hospitals
H. Use drugs with similar effect

Q23. What kinds of oral drugs did you still take during the epidemic? (Multiple choices)

A. Azole antifungal agent
B. Garlicin Capsule
C. Ganciclovir
D. Valaciclovir
E. Entecavir
F. Corticosteroid
G. Cyclosporine
H. Tacrolimus
I. Mycophernolate mofetil
J. Ruxolitinib
K. Imatinib
L. Dasatinib
M. Ibrutinib

Q24. Did you stop taking oral drugs due to difficulty in buying drugs during the epidemic?

A. Yes

B. No

Q25. What kinds of laboratory examinations did you still check-up regularly during the epidemic? (Multiple choices)

A. Blood routine

B. Biochemical function

C. Bone Marrow aspiration

D. Serum drug concentration

Q26. What kinds of laboratory examinations about COVID-19 did you take during the epidemic?

A. Severe acute respiratory syndrome coronavirus 2 (SARS-CoV-2) Nucleic acid detection

B. SARS-CoV-2 Antibody test

C. Both

D. Neither

Q27. Did you have relatives or friends who had been infected with COVID-19 during the epidemic?

A. Yes

B. No

Q28. What were you most worried about during the epidemic?

A. Go to hospital for adjusting treatment

B. Reexamination regularly

C. Have difficulty in buying drugs

D. Infection

E. GVHD

F. Relapse

G. Treatment conflict and prognosis

H. Go out

I. Go to school/ Work

J. Diet

K. Sleep

L. None

Questionnaire on the condition of hematopoietic stem cell transplantation recipients before COVID-19 outbreak

Q1. What is your name?

Q2. How old are you?

Q3. What is your gender?

A.Male

B.Female

Q4. Where did you live before the outbreak of coronavirus disease 2019(COVID-19)?

A.Wuhan city

B.Other cities in Hubei Province except Wuhan

C.Outside the Hubei province

Q5. What is your telephone number?

Q6. What is your primary disease?

I. Acute myeloid leukemia(AML)

J. Acute lymphoblastic leukemia(ALL)

K. Severe aplastic anemia(SAA)

L. Myelodysplastic syndrome(MDS)

M. Lymphoma

N. Chronic myeloid leukemia(CML)

O. Multiple myeloma(MM)

P. Others

Q7. Which blood group did you have before the hematopoietic stem cell transplantation (HSCT)?

Q8. When did you get the HSCT?

Q9. Which type of HSCT did you get?

C. Allogenic hematopoietic stem cell transplantation (Allo-HSCT)

D. Autologous hematopoietic stem cell transplantation (Auto-HSCT)

Q10. What is the relationship between the donor and you?

E. Parents

F. Brothers or sisters

G. Children

H. Unrelated donor (from Chinese Bone Marrow bank)

Q11. Which blood group did you have after the HSCT?

Q12. How many points in HLA- matching did you have?

Q13. Did you have graft-versus-host disease(GVHD)?

C. Yes

D. No

Q14. If you had GVHD, which organs were involved? (Multiple choices)

J. Skin

K. Mouth

L. Lungs

M. Liver

N. Gastrointestinal

O. Joints and fascia

P. Muscle

Q. Eyes

R. Others

Q15. Did you have a relapse of the primary disease before the COVID-19 epidemic?

C. Yes

D. No

Q16. Did you go out before the COVID-19 epidemic?

C. Yes

D. No

Q17. Did you wear mask consciously before the COVID-19 epidemic?

C. Yes

D. No

Q18. Did you live in a separate room before the COVID-19 epidemic?

C. Yes

D. No

Q19. Did you have the following symptoms before the COVID-19 epidemic? (Multiple choices)

D. Fever

E. Cough

F. Pulmonary infection (chest image)

Q20. When you had above symptoms, what did you do before the COVID-19 epidemic?

E. Go to the hospital
F. Take oral drugs by yourself
G. Consult doctor online/ telephone
H. None

Q21. Did you have difficulty in buying oral drugs before the COVID-19 epidemic?

C. Yes
D. No

Q22. How did you solve the difficulty in buying drugs before the COVID-19 epidemic?

I. Look for volunteers to buy drugs
J. Reduce the dose of oral drugs or stopping taking medicine
K. Buy drugs online
L. Borrow drugs from other patients
M. Buy drugs in pharmacies
N. Seek help from local government departments
O. Buy drugs in hospitals
P. Use drugs with similar effect

Q23. What kinds of oral drugs did you still take before the COVID-19 epidemic? (Multiple choices)

N. Azole antifungal agent
O. Garlicin Capsule
P. Ganciclovir
Q. Valaciclovir
R. Entecavir
S. Corticosteroid
T. Cyclosporine
U. Tacrolimus
V. Mycophernolate mofetil
W. Ruxolitinib
X. Imatinib
Y. Dasatinib
Z. Ibrutinib

Q24. Did you stop taking oral drugs due to difficulty in buying drugs before the COVID-19

epidemic?

C. Yes

D. No

Q25. What kinds of laboratory examinations did you check-up regularly before the COVID-19 epidemic? (Multiple choices)

E. Blood routine

F. Biochemical function

G. Bone Marrow aspiration

H. Serum drug concentration
